# Whole-Genome Sequence of the Fruiting Myxobacterium *Cystobacter fuscus* DSM 52655

**DOI:** 10.1128/genomeA.01196-17

**Published:** 2017-10-26

**Authors:** Anke Treuner-Lange, Marc Bruckskotten, Oliver Rupp, Alexander Goesmann, Lotte Søgaard-Andersen

**Affiliations:** a Department of Ecophysiology, Max Planck Institute for Terrestrial Microbiology, Marburg, Germany; b Bioinformatics and Systems Biology, Justus-Liebig University Giessen, Giessen, Germany

## Abstract

Among myxobacteria, the genus *Cystobacter* is known not only for fruiting body formation but also for formation of secondary metabolites, such as cystobactamids and cystothiazols. Here, we present the complete genome sequence of the *Cystobacter fuscus* strain DSM 52655, which comprises 12,349,744 bp and 9,836 putative protein-coding sequences.

## GENOME ANNOUNCEMENT

Members of the *Myxococcales* order provide a rich source of secondary metabolites with antibacterial and cytotoxic activities (1). Many *Myxococcales* species also have the capacity to generate spore-filled fruiting bodies in response to starvation (2–4). Interestingly, these fruiting bodies have very different morphologies (2). For instance, the model organism *Myxococcus xanthus* forms haystack-shaped cell aggregates (2). In contrast, the fruiting bodies of *Cystobacter fuscus* are shiny spherical sporangioles often clustered in groups within a gelatinous slime matrix (2). Little is known about the genetic basis underlying these morphological differences; however, comparative genome investigations of up to 10 different *Myxococcales* genomes have indicated that the developmental program leading to fruiting body formation and sporulation may not be highly conserved (5–7).

So far, 20 complete genomes and 36 draft genomes from members of the *Myxococcales* order are available (5, 8–32). To generate additional resources for accurate genome comparisons, we sequenced and annotated the complete genome of *Cystobacter fuscus* DSM 52655, which was obtained from Deutsche Sammlung von Mikroorganismen und Zellkulturen GmbH.

After confirming the formation of brown sporangioles clustered within a light brownish slime capsule by *C. fuscus* DSM 52655, we collected genomic DNA (33) and sequenced it using PacBio single-molecule real-time (SMRT) sequencing (34) on the PacBio RSII platform at the Max Planck Genome Centre, Cologne, Germany. Six SMRT-cells were used. Additionally, 12,193,527 100-bp paired-end Illumina reads were obtained using the HiSeq2000 platform. After quality evaluation and filtering of 188,247 PacBio subreads, the assembly process using the HGAP assembly pipeline (35) resulted in one contig with 62-fold coverage. The final contig was inspected manually using the Gepard dotplot generator (36) and YASS (37), manually closed using Illumina reads and the Pilon tool (38), and oriented to DnaA as the first locus tag. Prokka (39) was used to generate the annotation. BLASTp searches against the RefSeq database were used to assign functional annotations and to identify frameshifts. The corresponding genes were removed from the annotation.

The complete genome sequence of *C. fuscus* DSM 52655 contains 12,349,744 bp with a GC content of 68.5%. A total of 9,836 protein-coding sequences (CDSs) were identified together with 67 tRNA genes and 12 rRNA operons. The *C. fuscus* genome is similar in size to the incomplete genomes of *C. fuscus* DSM 2262 (GCA_000335475.2) and *C. ferrugineus* Cbfe23 (GCA_001887355.1) with sizes of 12.3 Mb and 12.1 Mb, respectively. Aligning the *C. fuscus* genome to other *Myxococcales* genomes using NUCmer (40) revealed overall synteny to the *C. fuscus* DSM 2262 and *C. ferrugineus* Cbfe23 genomes, with 79.5% and 75.6%, respectively, of the sequences aligning. The next best match found was to *Archangium gephyra* DSM 2261 with ~36% of the *C. fuscus* DSM 52655 genome aligning.

The *C. fuscus* DSM 52655 genome sequence provides a resource for the identification of gene clusters encoding enzymes involved in secondary metabolite synthesis and will ultimately help to understand the genetic basis underlying differences in fruiting body morphology.

### Accession number(s).

This genome sequence was deposited in GenBank under accession number CP022098.
